# Potentiality and Boundaries of Use of Sorafenib in Patients with Hepatocellular Carcinoma: Awaiting the Results of Ongoing Clinical Trials

**DOI:** 10.3390/cancers4020549

**Published:** 2012-06-05

**Authors:** Massimo Di Maio, Gennaro Daniele, Maria Carmela Piccirillo, Pasqualina Giordano, Giuseppe Signoriello, Bruno Daniele, Francesco Perrone

**Affiliations:** 1 Clinical Trials Unit, National Cancer Institute—“G. Pascale” Foundation, via Mariano Semmola, Napoli 80131, Italy; E-Mails: dimaiomax@libero.it (M.D.M.); gennaro.daniele@usc-intnapoli.net (G.D.); marilina.piccirillo@usc-intnapoli.net (M.C.P.); pasqualina.giordano@usc-intnapoli.net (P.G.); 2 Medical Statistics, Second University, v. L. Armanni 5, Napoli 80138, Italy; E-Mail: giuseppe.signoriello@unina2.it; 3 Medical Oncology Unit, “G.Rummo” Hospital, Benevento 82100, Italy; E-Mail: b.daniele@libero.it

**Keywords:** hepatocellular carcinoma, sorafenib, Child-Pugh B patients, trans-arterial chemo-embolization

## Abstract

No systemic therapy had been proven effective in patients with advanced hepatocellular carcinoma (HCC) until 2007, when a large randomized trial with sorafenib demonstrated a clinically relevant prolongation of survival. Currently, sorafenib represents standard treatment for patients with advanced HCC and well-preserved liver function, whilst the evidence about its effectiveness in patients with more severe liver impairment is less robust. A randomized trial to demonstrate the efficacy of sorafenib in Child-Pugh B patients with advanced HCC is currently ongoing. In the meantime, several trials are testing the role of sorafenib in early HCC (as adjuvant treatment after potentially curative loco-regional therapies) and in intermediate stage (exploring different modalities of integration of sorafenib with trans-arterial chemo-embolization). The results of all these trials will better define the potentiality and the boundaries of use of sorafenib in HCC patients.

## 1. Introduction

**Figure 1:**
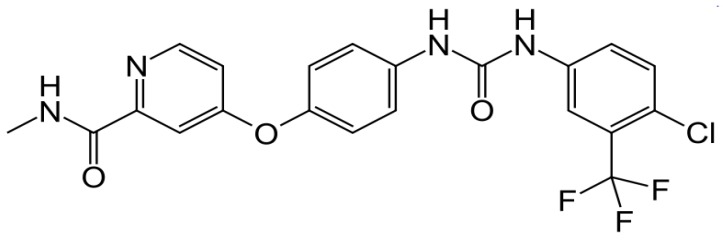
Chemical structure of sorenafib.

Sorafenib (Nexavar^®^, Bayer/Onyx Pharmaceuticals, Figure 1) is an orally active inhibitor of multiple receptor tyrosine kinases involved in pathways relevant for tumor growth and angiogenesis, including Raf 1, B-Raf, vascular endothelial growth factor receptor (VEGFR)-1, VEGFR-2, VEGFR-3, and platelet-derived growth factor receptor β [1]. Hepatocellular carcinoma (HCC) is a tumor that typically shows a high level of vascularization, and both VEGF and PDGF have been implicated in tumor angiogenesis of HCC [2,3]. Cellular signaling that is mediated by both the Raf-1 and VEGF pathways has been implicated in the molecular pathogenesis of HCC, providing a strong rationale for testing sorafenib in this setting. In preclinical experiments, sorafenib showed antiproliferative activity in liver cancer cell lines, and it reduced tumor angiogenesis and increased tumor cell apoptosis in a mouse xenograft model of human HCC [4].

The drug, already approved for the treatment of patients with advanced renal cell carcinoma, showed very promising results in a phase 2 trial with 137 patients with advanced hepatocellular carcinoma (HCC), although the study was formally negative in terms of objective response rate classically measured [5]. The SHARP study [6] was a randomized phase 3 trial comparing sorafenib (at the dose of 400 mg twice daily) *versus* placebo in patients with advanced HCC. The use of placebo for patients assigned to control arm was ethically and scientifically justified by the absence of pharmacological treatments with proven efficacy in this setting. In the trial, which included a population of patients with relatively preserved liver function (Child-Pugh class A), the use of sorafenib was associated, at the second planned interim analysis, with a statistically significant and clinically relevant benefit in terms of survival over placebo (median 10.7 months *versus* 7.9 months; hazard ratio [HR] 0.69; 95% confidence interval [CI] 0.55–0.87; *p* < 0.001). On the basis of these results, sorafenib was approved for the treatment of HCC by the European Agency for the Evaluation of Medicinal Products (EMA) in October 2007 and by the U.S. Food and Drug Administration (FDA) in November 2007. Subsequently, a similar randomized phase 3 comparison of sorafenib *versus* placebo was conducted in Asian patients with Child-Pugh class A [7], showing a similar benefit in terms of survival in 271 patients with advanced HCC (HR 0.68, 95% CI 0.50−0.93, *p* = 0.014).

## 2. Boundaries of the Use of Sorafenib

### 2.1. From Clinical Trials to Clinical Practice: When to Start Sorafenib?

Randomized trials documenting the efficacy of sorafenib were conducted in patients judged to be ineligible (or no longer eligible) for loco-regional treatment. To date, “precocious” use of sorafenib in patients who are theoretically eligible for loco-regional treatment would represent an under-treatment, and should be carefully avoided. On the other hand, a clear definition of failure of loco-regional treatment would help avoiding further, potentially ineffective local approach and inappropriate delay of systemic treatment. From this point of view, a multi-disciplinary evaluation of each candidate to systemic treatment will confirm the absence of eligibility for a loco-regional approach. In recent years, prompted by the availability of an effective systemic treatment, efforts have been made to better define criteria of failure and contra-indications to administration of trans-arterial chemoembolization [8].

### 2.2. From Clinical Trials to Clinical Practice: When to Stop Sorafenib? Use of Sorafenib Beyond Progression

In the SHARP registrative trial, sorafenib was administered, in the absence of unacceptable toxicity, until the occurrence of both radiologic progression, as defined by RECIST criteria, and symptomatic progression, as defined by the Functional Assessment of Cancer Therapy-Hepatobiliary Symptom Index 8 questionnaire [6]. In other words, in the presence of documented instrumental progression but in the absence of symptomatic worsening, a patient continued the drug. This was conceptually allowed by the absence of effective second-line treatments, and also by the sub-optimal performance of RECIST dimensional criteria in advanced HCC, with potential discrepancy between definition of progressive disease by RECIST and treatment activity in terms of tumor necrosis [9]. Furthermore, a prolongation of time to symptom deterioration was a co-primary endpoint of the trial, together with overall survival. Unfortunately, however, this endpoint was not met, because there was no difference between sorafenib and placebo in terms of time to symptom worsening. Median time to symptomatic progression was equal to 4.1 months with sorafenib *versus* 4.9 months with placebo (HR 1.09, 95% CI 0.88–1.31, *p* = 0.77) [6]. Similarly, negative results in terms of time to symptom deterioration were observed in the Asia-Pacific trial: median time to symptom deterioration was equal to 3.5 months with sorafenib *versus* 3.4 months with placebo (HR 0.90, 95% CI 0.67–1.22, *p* = 0.50) [7].

Recently, several experts have suggested the possibility of continuing sorafenib beyond disease progression [10]. In a consensus statement about the use of sorafenib for the treatment of advanced HCC, Peck-Radosavljevic and colleagues suggest that, in the absence of alternative therapies with proven efficacy in this setting, continuing sorafenib treatment after disease progression may be beneficial in slowing down tumour growth [10]. According to their opinion, sorafenib may be continued after disease progression for patients with stable performance status, although they recognize that there is currently no clear evidence supporting the effectiveness of this approach. We believe that, in the absence of a clear proof of efficacy in this setting, sorafenib should be not be administered beyond progression. Although well tolerated, sorafenib could be associated with toxic effects and “first, do not harm” should be the main rule to follow for the optimal treatment of patients in clinical practice.

Recently, a randomized phase II trial explored the efficacy of sorafenib beyond progression [11]. Briefly, 101 patients, out of 300 prospectively treated with first-line sorafenib at standard dose, were randomized at the time of documented radiological progression to increased-dose sorafenib (600 mg twice daily) plus best supportive care (BSC) or to BSC alone. Median progression-free survival was equal to 3.9 months in patients assigned to sorafenib and 2.7 months in patients assigned to BSC (HR 0.67, 95% CI 0.43–1.06, *p* = 0.089). Similarly, median time-to-progression was equal to 3.97 months in patients assigned to sorafenib and 2.0 months in patients assigned to BSC (HR 0.59, 95% CI 0.33–1.05, *p* = 0.07). At last, median overall survival was equal to 7.55 months in patients assigned to sorafenib and 5.98 months in patients assigned to control arm (HR 0.71, 95% CI 0.47–1.08, *p* = 0.107). According to the authors, also in light of the acceptable tolerability profile, these results are promising, although not statistically significant, and suggest that increased-dose sorafenib may be used as a control arm in phase II–III trials evaluating new drugs as second line therapy in HCC. In our opinion, despite the interesting *rationale*, study results are not sufficient to demonstrate any advantage supporting sorafenib continuation beyond progression, and evidence-based approach suggests that best supportive care should remain the treatment for patients assigned to control arm in randomized trials of second-line treatments. At the best of our knowledge, there are no other ongoing trials testing this strategy. An improvement in the treatment options for patients experiencing progression during sorafenib will probably come from better knowledge of mechanisms of tumor resistance, and from the availability of effective second-line treatment options [12]. A phase 3 randomized trial comparing brivanib *versus* placebo as second line treatment (BRISK-PS, Brivanib Study in HCC Patients at Risk Post Sorafenib, Clinicaltrials.gov Identifier NCT00825955) has been completed. Detailed data are still not published, but, at the end of 2011, a press release from the manufacturer of brivanib, revealed that in the phase 3 trial brivanib “did not meet the primary endpoint of improving overall survival *versus* placebo” [13].

### 2.3. External Validity of the Randomized Trials Testing Sorafenib in Advanced HCC

Both the SHARP trial [6] and the analogue trial in the Asian patients [7] enrolled only patients with advanced HCC and preserved liver function (Child-Pugh class A). In clinical practice, a relevant proportion of patients with advanced HCC suffer from worse impairment of liver function. Several years after the publication of the two registrative trials, however, the effectiveness of sorafenib in Child-Pugh B patients remains an open question. This lack of knowledge was ignored by regulatory bodies, as both European Medicines Agency (EMA) and US Food and Drug Administration (FDA) approval did not take into account liver function. In detail, FDA approved sorafenib with a broad indication for the treatment of unresectable HCC, without limitation in terms of Child-Pugh category, considering the paucity of treatment options and heterogeneity of patients within a given Child-Pugh class, to facilitate clinical judgment for individual patients [14]. Registration by EMA was even more generic, because it is not specifically limited to advanced setting. Thus, according to registration, HCC patients with Child-Pugh B can receive sorafenib; to date, however, this indication is not supported by the results of any randomized clinical trial.

Until recently, the only published data on the safety of sorafenib and outcome in patients with compromised liver function came from a small cohort of Child-Pugh B patients enrolled in the initial phase II trial [15], and from some case series [16,17]. The Global Investigation of Therapeutic DEcisions in Hepatocellular Carcinoma and Of its Treatment with SorafeNib (GIDEON) study (ClinicalTrials.gov identifier NCT00812175) was a global, prospective, non-interventional study of patients with unresectable HCC, eligible for systemic therapy and for whom the decision to treat with sorafenib had been taken under real-life practice conditions [18]. The declared aim of the GIDEON study was to evaluate the safety and efficacy of sorafenib in different subgroups, especially Child-Pugh B where data are limited. Preliminary results of the study have produced quite reassuring data about the tolerability of the drug in Child-Pugh B patients [19]. In detail, at the second interim safety analysis, based on approximately half of the 3,322 patients enrolled patients, 957 subjects were classified as Child-Pugh A and 367 as Child-Pugh B. Adverse events rate was indeed similar among the groups, but serious events occurred in 56% of Child-Pugh B patients, compared to 29% of Child-Pugh A patients, and drug-related serious adverse events were seen in 15%, and 8%, respectively. As expected, median duration of treatment was shorter in Child B patients (9 *versus* 14 weeks) and 46% of Child B patients stopped treatment in the first 8 weeks, compared to 30% of Child A patients. Authors concluded that preliminary results indicate that the sorafenib safety profile is generally similar in the Child-Pugh B and Child-Pugh A patients. However, despite the results of the GIDEON study are relevant because they increase the amount of available data regarding the use of sorafenib in Child B patients, the real efficacy of the drug in these patients remains unknown, due to the lack of randomized controlled trials [20,21]. Given this status, two different approaches are possible in clinical practice [20]. On one hand, physicians might decide to apply “methodological purism”, strictly considering the external validity of randomized trial results and denying the drug to patients who do not meet eligibility criteria. With this approach, treatment of Child-Pugh B HCC patients remains an unmet need, due to the absence of alternative treatments, and no therapeutic chance is offered to these patients. On the other hand, physicians might decide to apply “clinical pragmatism”, taking into account the absence of therapeutic alternatives, and offering sorafenib to Child-Pugh B patients.

Child-Pugh B patients represent a population whose prognosis is strictly affected not only by the presence of HCC, but also by the compromised liver function. In these patients, even assuming no detrimental impact of sorafenib in terms of toxicity and deterioration of liver function, the real impact of anti-tumor efficacy of sorafenib on patients’ outcome can be significantly “diluted” by the competing risk of death (liver failure). Randomized registrative trials were conducted in a setting of patients with well preserved liver function, where cancer survival is less confounded by deaths due to liver failure. In our opinion, this appears to be closer to a so-called “exploratory” approach (aimed at understanding the “true” effect of the drug in close-to-ideal conditions), more than a “pragmatic” approach (aimed at clinical decisions, *i.e*., is sorafenib really effective and worth to be used in patients with impaired liver function?) [22]. The discussion about the effectiveness of sorafenib in patients with impaired liver function should actually shift from the theoretical question “Can it work?” to the practical questions “Does it work?” and “Is it worth?” under real-life conditions [23].

**Figure 2:**
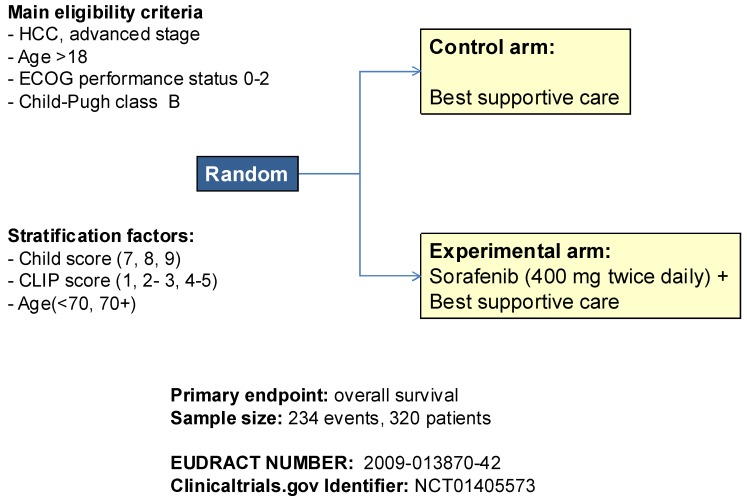
Design of the BOOST randomized phase III trial, comparing sorafenib + best supportive care (BSC) *versus* BSC alone in Child-Pugh B patients with advanced HCC.

In order to verify, according to a pragmatic approach, whether sorafenib is effective also in Child-Pugh B patients, a non-profit, academic study, called BOOST (B Child HCC patients—Optimization of Sorafenib Treatment), has been designed [24]. BOOST (ClinicalTrials.gov Identifier NCT01405573; Eudract number 2009-013870-42) is a randomized phase 3 trial comparing sorafenib + best supportive care (BSC) *versus* BSC alone in Child-Pugh B patients with advanced HCC (Figure 2). Patients are eligible in the trial if older than 18, with ECOG performance status 0–2. Patients assigned to experimental arm receive sorafenib 400 mg twice daily, with planned dose reductions and treatment interruptions according to adverse events. The study is designed with overall survival as primary endpoint. The sample size is calculated in order to demonstrate a Hazard Ratio of death 0.70 in favor of sorafenib (similar to the HR demonstrated in the SHARP trial, although corresponding to a smaller improvement in median OS, from 4.5 to 6.5 months, given the worse prognosis of Child-Pugh B patients). Overall, with 80% statistical power and α equal to 0.05, 320 patients have to be randomized, 160 assigned to sorafenib and 160 assigned to supportive care alone.

The BOOST trial is already approved by the Ethical Committee of the National Cancer Institute, Napoli, Italy, as coordinating centre, and is currently under evaluation by several other Institutions. Given the current approval of sorafenib in both the U.S. and Europe, that is unrestricted in terms of liver function, the BOOST trial is reasonably out of interest for the company producing the drug. The trial has received a partial financial support by Italian Ministry of Health. We strongly believe that the results of the BOOST study would be in any case important. A positive result would produce the first robust, convincing evidence for treating Child-Pugh B patients with sorafenib. On the other hand, a negative result would of course spare potential toxicity to a category of frail patients. Both positive and negative results might have effects on regulatory decisions regarding label and reimbursement of sorafenib, both in Italy (where the drug is currently reimbursed only for Child-Pugh A patients) and outside.

## 3. Potentialities of the Use of Sorafenib

### 3.1. Experimental Use of Sorafenib in Patients with Early Stage HCC

A relevant proportion of patients, diagnosed with early stage HCC and successfully treated with surgical resection or local ablation therapies, will subsequently experience tumor relapse. Although several clinical trials have been conducted to test the efficacy of adjuvant treatments following surgical resection or complete necrosis obtained with ablation, there is no systemic treatment with established efficacy in this setting. A randomized phase III study, the STORM trial (Clinicaltrials.gov Identifier NCT00692770), comparing sorafenib to placebo as adjuvant treatment for patients who have received surgical resection or local ablation, without evidence of residual disease, is ongoing [25]. To be eligible, patients had to be performance status 0 according to ECOG scale, and a well-preserved liver function (Child-Pugh score between 5 and 7). Primary endpoint of the trial is recurrence-free survival. The STORM trial has already reached its final sample size (approximately 1,100 patients), but results are still awaited. Final data collection for primary outcome measure is estimated for the end of 2012.

### 3.2. Experimental Use of Sorafenib in Patients with Intermediate Stage HCC

Transcatheter arterial chemo-embolization (TACE) is the current standard of treatment for patients with intermediate stage HCC [26]. According to current guidelines, TACE should be considered in fit patients with inoperable HCC, for solitary, large nodules or for multifocal HCC [26]. Patients grouped into intermediate stage HCC, however, represent a very heterogeneous population, in terms of tumor burden and liver function [26]. Although selective and super-selective delivery of drugs in arterial branches is able to reduce the toxic impact on non-tumoral liver, treatment is necessarily limited to patients with good performance status and well preserved hepatic function. The body of evidence about the efficacy of TACE has been almost exclusively produced with conventional procedure, based on a selective access to the vascular bed of the tumor allowing both vessel embolization and intratumoural infusion of anti-cancer drugs like doxorubicin. In recent years, promising data in terms of activity and tolerability have been obtained using doxorubicin-loaded beads rather than the conventional doxorubicin-lipiodol emulsion [27].

Several randomized trials are testing the efficacy of sorafenib in combination with TACE for patients with intermediate stage HCC. There is an interesting rationale for potential synergy between the two treatment strategies [28]. In a study of tumor specimens from patients with HCC treated with TACE or with surgical resection, the number of VEGF-positive cells in the TACE group was higher than in samples from patients treated with surgery alone [29]. The explanation of this finding is related to the results of another experimental model, based on the administration of TACE to liver tumors grown in the livers of New Zealand white rabbits [30]. In this setting, hypoxia caused by the procedure determined a significant increase in hypoxia-inducible factor-1alpha, a transcription factor that in turn regulates other pro-angiogenic factors. In a preclinical model in rats with liver cancers undergoing transarterial embolization (TAE), intraportal injection of recombinant adeno-associated virus vector encoding rat angiostatin inhibited the angiogenesis stimulated by TAE, synergized with TAE in suppressing growth of tumors established in livers and prolonged the survival of rats [31]. Based on this laboratory evidence, the anti-angiogenic activity of sorafenib, together with its well-established efficacy in the setting of advanced disease, represents an intriguing *rationale* for testing its addition to TACE. In principle, it has been emphasized that, if the relative magnitude of the benefit associated with the use of sorafenib in the intermediate stage setting were the same observed in advanced disease (Hazard Ratio 0.69 in the SHARP randomized trial), the net survival improvement in patients receiving TACE plus sorafenib might reach 6 months [32].

On a theoretical basis, administration of sorafenib can be combined to the loco-regional procedure in at least three different ways: a sequential schedule, an interrupted schedule, a continuous schedule [33]. In a sequential schedule, sorafenib is administered after the completion of the loco-regional treatment. In an interrupted schedule, sorafenib is stopped before each session of TACE, and resumed after the procedure took place. In a continuous schedule, patients assume sorafenib continuously, without interruption in the days of loco-regional procedure.

In the sequential schedule, sorafenib is used like an adjuvant treatment, with the aim of treating tumor cells that have escaped the local treatment, preventing the re-growth of treated lesions and eventually the growth of new tumor lesions [33]. Of course, this schedule has the advantage of avoiding potential addition of toxicities, but it does not allow the treatments to interact synergistically. Unfortunately, results obtained in the Asian study with sequential administration of sorafenib after a response with TACE have been disappointing [34]. Patients from South Korea or Japan, with unresectable HCC, well-preserved liver function and at least 25% tumor necrosis or shrinkage after one or two TACE, were randomized to receive sorafenib (n = 229), at the standard dose of 400 mg twice daily, or placebo (n = 229). The primary aim of the trial was to demonstrate a prolongation of time to progression, but there was no statistically significant difference between the arms: median time to progression was 5.4 and 3.7 months, respectively (HR 0.87, 95% CI 0.70–1.09, *p* = 0.252). Despite the sequential schedule, there was a quite unexpected high incidence of side-effects reported with sorafenib in this study population: 73% of the patients had dose reductions, and 91% had treatment interruption. Due to poor treatment tolerability, dose intensity was lower than expected: median daily dose of sorafenib was 386 mg/day, lower than the half of theoretical full dose. In their discussion, authors underline that, due to study procedures that needed centralized response confirmation, more than 50% of the patients started treatment with sorafenib later than 9 weeks after the completion of loco-regional procedure, and this could have compromised the efficacy of the combined strategy.

Whether the negative results of the study have been really conditioned by the sequential rather than concomitant administration of treatments is unknown. However, the majority of the other ongoing trials in this setting are testing the concomitant (interrupted or continuous) administration of sorafenib with the loco-regional treatment, in order to maximize the potential synergistic effect of the two approaches. Concomitant administration could potentially be associated with an increase in treatment-related adverse events, especially considering that toxicity was not negligible even in the sequential schedule. However, reassuring results come from a recently published phase I trial [35], testing the continuous administration of sorafenib in 14 patients, with dose escalation from 200 mg twice daily to 400 mg twice daily, starting 7 days prior to TACE with doxorubicin. Because there were no dose-limiting toxicities in the first three patients who received sorafenib at a dose of 200 mg twice daily, all the remaining patients received the full standard dose of the drug. Twenty-seven procedures were performed (median, two per patient), and the experimental schedule was proven tolerable: the adverse event profile of this regimen was comparable with that of sorafenib monotherapy (hand-foot skin reaction, weight loss, diarrhea, abdominal pain), with the exception of thrombocytopenia, which may be more frequent (three patients experienced severe thrombocytopenia). Of note, there were no increases in the circulating VEGF levels after TACE combined with sorafenib. After combined treatment, there was a significant decrease in the concentration of plasma VEGF.

Several trials have been designed to test the concomitant (interrupted or continuous) addition of sorafenib to TACE in patients with intermediate stage HCC. In a prospective, single-arm phase II study in 35 patients with unresectable HCC, sorafenib was administered at standard dose (400 mg twice daily) combined with doxorubicin-eluting beads TACE [36]. Sorafenib administration was initiated 1 week before TACE, and the drug was administered continuously. For the first 11 patients in the study, a sorafenib dose interruption (3 days before and after TACE) was utilized, because no clinical data were available on the safety of combination therapy. Only after an early analysis, demonstrating a good safety profile of combination therapy, with no increase in grade 3 to 4 toxicity as compared with existing data on patients treated with TACE or sorafenib alone, all subsequent patients were allowed to receive sorafenib continuously throughout the treatment cycles. The most common toxicities during cycle one were fatigue (94%), anorexia (67%), alterations in liver enzymes (64%), and dermatologic adverse effects (48%). Although most patients experienced at least one grade 3 to 4 toxicity, most toxicities were mild or moderate in intensity. Activity data are promising, but the real role of addition of sorafenib to loco-regional procedure was difficult to evaluate, due to the absence of a control arm. The SPACE study is a randomized phase II trial, designed to give a preliminary evaluation of the efficacy and safety of the addition of sorafenib to TACE (using doxorubicin-eluting beads) compared to TACE plus placebo in patients with intermediate-stage HCC (BCLC B, defined as the presence of asymptomatic, unresectable, multinodular tumors without vascular invasion or extrahepatic spread) and Child-Pugh class A status without ascites [37]. Eligible patients undergoing TACE with doxorubicin-eluting beads (loaded with 150 mg doxorubicin) were randomized to sorafenib (standard dose, 400 mg twice daily) or placebo, on a continuous basis. Treatment cycles were repeated every 4 weeks until untreatable progression (defined as failure to achieve objective response after ≥2 TACE in the treated tumor nodule). TACE is performed on day 1 of cycles 1 (3–7 days after first dose of sorafenib), 3, 7, 13, and every 6 cycles thereafter. The primary study endpoint was time to progression, with secondary endpoints overall survival, time to untreatable progression, time to vascular invasion/extrahepatic spread, and safety. Overall, 307 patients were randomized in the trial (154 patients in the sorafenib group and 153 assigned to placebo). Median treatment duration was shorter in the experimental arm: 4.8 months in the sorafenib group *versus* 6.3 months in the placebo group [38]. The objective response rate (35.7% *versus* 28.1%) and rate of progressive disease (13% *versus* 23.5%) both favored the sorafenib arm. However, median time-to-progression was nearly identical in the two arms: 169 days with sorafenib *versus* 166 days for patients assigned to placebo, although the study was formally positive given the predefined, exploratory alpha of 0.15 (HR = 0.797; 95% CI, 0.588–1.080, *p* = 0.072). There was no difference in overall survival (HR = 0.898; 95% CI, 0.606–1.330, *p* = 0.295). Authors emphasized that no new safety findings that would discourage the use of sorafenib in this combination were observed, and that the above described results represent, in their opinion, positive signals that need to be confirmed in ongoing phase 3 trials in this patient population.

In a randomized phase III trial currently performed by Eastern Cooperative Oncology Group (ClinicalTrials.gov Identifier NCT01004978), patients with unresectable HCC, Child-Pugh class A or B7, are assigned to control arm (TACE alone) or experimental arm (TACE plus sorafenib at standard dose, until disease progression or unacceptable toxicity) [39] (Table 1). In the experimental arm, patients start treatment with sorafenib and, within 2 weeks after a stable dose of sorafenib is reached, patients undergo TACE. Several TACE techniques are allowed by the protocol: doxorubicin hydrochloride, mitomycin C, and cisplatin (closed to accrual in 2010); conventional chemoembolization comprising doxorubicin hydrochloride only; or chemoembolization comprising doxorubicin-eluting beads. Treatment with TACE is repeated every 4 weeks, for up to 4 courses. Primary endpoint of the trial is progression-free survival. Planned accrual is 400 patients, and the trial should be completed by the end of 2012.

**Table 1:** Ongoing randomized trials testing the addition of sorafenib to trans-arterial chemoembolization for patients with intermediate stage hepatocellular carcinoma.

Trial (ClinicalTrials.gov Identifier)	Type of trial	Treatment arms	Planned number of patients	Expected time for results
Eastern Cooperative Oncology Group (NCT01004978) [39]	Phase III	**Experimental arm**: Patients receive oral sorafenib tosylate twice daily in the absence of disease progression or unacceptable toxicity. Beginning within 2 weeks after a stable dose of sorafenib tosylate is reached, patients undergo TACE comprising doxorubicin hydrochloride, mitomycin C, and cisplatin (closed to accrual in 2010); conventional chemoembolization comprising doxorubicin hydrochloride only; or chemoembolization comprising doxorubicin-eluting beads. Treatment with TACE repeats approximately every 4 weeks for up to 4 courses in the absence of disease progression or unacceptable toxicity. **Control arm**: Patients receive oral placebo twice daily in the absence of disease progression or unacceptable toxicity. Beginning within 2 weeks after a stable dose of placebo is reached, patients undergo TACE as in experimental arm.	400	September 2012
University College of London TACE-2 (NCT01324076) [40]	Phase III	**Experimental arm**: Patients receive oral sorafenib tosylate twice daily in the absence of disease progression or unacceptable toxicity. Beginning within 2–5 weeks after start of sorafenib tosylate, patients undergo TACE with doxorubicin-eluting beads. Patients may undergo additional sessions of TACE with doxorubicin-eluting beads, in the absence of complete devascularization of the tumor(s).	412	November 2014
University College of London TACE-2 (NCT01324076) [40]	Phase III	**Control arm**: Patients receive oral placebo twice daily in the absence of disease progression or unacceptable toxicity. Beginning within 2–5 weeks after start of placebo, patients undergo TACE with doxorubicin-eluting beads. Patients with disease progression may cross over to the sorafenib tosylate arm at the discretion of the treating clinician.	412	November 2014
Japan Liver Oncology Group TACTICS (NCT01217034) [41]	Phase II	**Experimental arm**: Sorafenib will be administrated at a dose of 400 mg o.d. before the first TACE. After 2 days drug rest, TACE will be conducted. Sorafenib will be resumed at a dose of 400 mg o.d. from 3 days after TACE(the resumption day can be postponed until 21 days after TACE). When tolerability is confirmed at 1 week after resumption, the dose of sorafenib will be increased to 400 mg b.i.d. When tumor increases, TACE will be repeated. **Control arm**: TACE will be conducted at scheduled day. When tumor increases, TACE will be repeated.	228	September 2016

Another randomized trial, conducted by the University College of London (Clinicaltrials.gov Identifier NCT01324076), is comparing sorafenib to placebo in addition to TACE with doxorubicin-eluting beads [40] (Table 1). Patients receive oral sorafenib or placebo twice daily until disease progression or unacceptable toxicity. Beginning within 2–5 weeks after start of systemic treatment, patients undergo TACE with doxorubicin-eluting beads, and may undergo additional sessions in the absence of complete devascularization of the tumor. Primary endpoint of the trial is progression-free survival, planned accrual is 412 patients, and the trial should be completed by the end of 2014.

Finally, the interrupted schedule of sorafenib in the days just before and after the administration of loco-regional therapy has the advantage of avoiding delays in the start of systemic treatments, like the continuous schedule, but is should avoid additional effects on toxicity as much as possible. Of course, the advantage in terms of safety could be outbalanced by the disadvantage related to interruption itself, producing sub-therapeutic levels of the drug in a potentially critical time-frame. Intermittent addition of sorafenib to TACE was tested in a single-arm phase II trial [42]. Sorafenib (standard dose, 400 mg twice daily) was started 4 days after the first TACE ad assumed continuously, with treatment-free holidays 4 days before and after each TACE procedure. Preliminary results of the trial, presented in 2010, were promising in terms of safety and activity, but the absence of a control arm does not allow robust conclusions about the efficacy of the tested schedule. A randomized phase II trial performed by the Japan Liver Oncology Group is testing the addition of sorafenib, administered in an intermittent schedule, to TACE (TACTICS trial, Clinicaltrials.gov Identifier: NCT01217034) [41] (Table 1). In the experimental arm, patients start sorafenib at reduced dose (400 mg every other day), before receiving the first TACE procedure. Sorafenib is interrupted 2 days before TACE, and resumed at the same reduced dose from 3 days after the loco-regional procedure. When tolerability is confirmed at 1 week after resumption, the dose of sorafenib is increased to standard dose (400 mg twice daily). Primary endpoint of the trial is time to untreatable progression, planned accrual is 228 patients, and the results should be available in 2016. At the moment, the addition of sorafenib to standard treatment of patients with intermediate HCC remains completely experimental, because there are no solid data supporting this strategy outside of clinical trials setting.

### 3.3. Experimental Use of Sorafenib in Combination with Other Treatments in Patients with Advanced HCC

Before the demonstration of efficacy of sorafenib, no systemic treatment for HCC had been associated with definite survival benefit [43]. This might partially depend on specific chemo-resistance of HCC due to the expression of the multi-drug resistance gene MDR-1, and partially to liver cirrhosis that is present in most patients, which prevents the administration of full dosage of many drugs. Among cytotoxic drugs, doxorubicin has been diffusely used as treatment for patients with advanced HCC, despite low response rates and a not negligible toxicity. In a phase II randomized trial, comparing sorafenib plus doxorubicin *versus* doxorubicin alone in patients with advanced HCC and compensated liver function (Child A), the addition of sorafenib to doxorubicin produced an improvement in both overall survival and progression-free survival [44]. However, as recognized by the authors of the trial, the absence of a control group treated with sorafenib alone precludes any assessment of the potential synergism between doxorubicin and sorafenib. In other words, the results of that trial do not explain whether the cytotoxic agent contributed significantly to the outcome or whether the benefit seen in the combination arm was the result of sorafenib alone. A randomized phase III trial is currently ongoing, sponsored by the Cancer and Leukemia Group B (ClinicalTrials.gov Identifier NCT01015833), testing the addition of doxorubicin to sorafenib. Patients assigned to standard arm receive sorafenib until disease progression, while patients assigned to experimental arm receive in addition doxorubicin for a maximum of six cycles. Primary endpoint of the trial is overall survival, and 480 patients are planned. At the moment, waiting for the results of this trial, the combination of sorafenib with doxorubicin or other cytotoxic agents must be considered experimental and cannot be proposed for clinical practice.

Although external beam radiation therapy can produce high local control rates in HCC patients, survival of patients treated with radiotherapy is limited by the high frequency of intra- and extra-hepatic recurrences. Radiotherapy is not included in current guidelines for the treatment of patients with advanced HCC. According to some authors, the combination of radiotherapy and sorafenib could be potentially useful, because the administration of the drug could target the RAF and VEGFR signaling pathways, which are specifically activated after exposure to radiation, and are responsible for radio-resistance. In addition, sorafenib could enhance the oxygen effect through normalization of the surviving tumor vasculature, and could determine synchronization of the cell cycle [45]. Based on this rationale, the combination of sorafenib and radiotherapy is currently being evaluated in phase I–II trials (ClinicalTrials.gov Identifiers NCT00892658, NCT01005875, NCT01319942, NCT01328223). Currently, this combination cannot be considered outside the context of clinical trials.

## 4. Conclusions

Currently, sorafenib represents standard treatment for patients with advanced HCC and well-preserved liver function. A multi-disciplinary evaluation of each patient candidate to systemic treatment should confirm the absence of eligibility for a loco-regional approach. To date, there is no robust evidence supporting sorafenib continuation beyond progression, and evidence-based approach suggests that best supportive care should remain the treatment for patients assigned to control arm in randomized trials of second-line treatments. Compared to patients with good liver function, the evidence about the effectiveness of sorafenib in patients with more severe liver impairment is less robust. A pragmatic phase III randomized trial is currently ongoing, to demonstrate the efficacy of sorafenib in Child-Pugh B patients with advanced HCC. In the meantime, several trials are testing the role of sorafenib in early HCC (as adjuvant treatment after potentially curative loco-regional therapies) and in intermediate stage (exploring different modalities of integration between sorafenib and trans-arterial chemo-embolization), in order to reproduce in these settings the benefit associated with the use of the drug in the advanced stage. The results of all these trials will better define the potentiality and the boundaries of use of sorafenib in HCC patients.
